# *Plasmodium falciparum *exposure *in utero*, maternal age and parity influence the innate activation of foetal antigen presenting cells

**DOI:** 10.1186/1475-2875-8-251

**Published:** 2009-11-05

**Authors:** Nadine Fievet, Stefania Varani, Samad Ibitokou, Valérie Briand, Stéphanie Louis, René Xavier Perrin, Achille Massougbogji, Anne Hosmalin, Marita Troye-Blomberg, Philippe Deloron

**Affiliations:** 1 UR010, Mother and Child Health in the Tropics, Institut de Recherche pour le Développement (IRD), Cotonou, Benin; 2 UR010, IRD, IFR 71 Université René Descartes, 75006 Paris, France; 3 Department of Immunology, Wenner-Gren Institute, Stockholm University, Stockholm, Sweden; 4 Department of Hematology and Oncology, "L. and A. Seragnoli" University of Bologna, Bologna, Italy; 5 Faculté des Science de la Santé, Cotonou, Benin; 6 Institut Cochin, Université Paris Descartes, CNRS (UMR 8104), Paris, France; 7 Inserm, U567, Paris, France

## Abstract

**Background:**

Malaria in pregnancy is associated with immunological abnormalities in the newborns, such as hampered T-helper 1 responses and increased T-regulatory responses, while the effect of maternal *Plasmodium falciparum *infection on foetal innate immunity is still controversial.

**Materials and methods:**

The immunophenotype and cytokine release by dendritic cells (DC) and monocytes were evaluated in cord blood from 59 Beninese women with or without malaria infection by using flow cytometry.

**Results:**

Accumulation of malaria pigment in placenta was associated with a partial maturation of cord blood myeloid and plasmacytoid DC, as reflected by an up-regulated expression of the major histocompatibility complex class II molecules, but not CD86 molecules. Cells of newborns of mothers with malaria pigment in their placenta also exhibited significantly increased cytokine responses upon TLR9 stimulation. In addition, maternal age and parity influenced the absolute numbers and activation status of cord blood antigen-presenting cells. Lastly, maternal age, but not parity, influenced TLR3, 4 and 9 responses in cord blood cells.

**Discussion:**

Our findings support the view that placental parasitization, as indicated by the presence of malaria pigment in placental leukocytes, is significantly associated with partial maturation of different DC subsets and also to slightly increased responses to TLR9 ligand in cord blood. Additionally, other factors, such as maternal age and parity should be taken into consideration when analysing foetal/neonatal innate immune responses.

**Conclusion:**

These data advocate a possible mechanism by which PAM may modulate foetal/neonatal innate immunity.

## Background

Pregnancy-associated *Plasmodium falciparum *malaria (PAM) results, sometimes, in massive intervillous inflammation that contributes to placental insufficiency, impaired intra-uterine growth and consequently to low birth weight in the newborns and a higher risk of dying early in life [1-4].

Infants born to women with PAM are more predisposed to *P. falciparum *infection in their first year of life [5-7]. Immunological mechanisms are generally considered to play an important role in causing this susceptibility. *In utero *sensitization to transplacentally transferred soluble *P. falciparum *antigens may constitute the basis for increased susceptibility to malaria episodes in early life. Importantly, it has been demonstrated that cord blood mononuclear cells (CBMC) of neonates born to mother with PAM specifically respond to plasmodial asexual stage antigens, and that cord blood B cells produce anti-malaria specific IgM and IgE antibodies [5,8-10], providing irrefutable evidence of *in utero *sensitization.

In this context, active infection in the placenta by *P. falciparum *was associated with hampered T-helper 1 (Th1) responses, as reflected by reduced IFN-γ production upon T-cell stimulation [9]. In addition, the anti-inflammatory IL-10 cytokine is more frequently produced by CBMC of those born to mothers with PAM compared with non-infected mothers [11]. CD4^+^CD25^high ^regulatory T-cells (Treg) are a principal source of IL-10 in such cases [12]. Treg are found at higher frequency in cord blood (CB) of neonates born to mothers with PAM at delivery as compared to unexposed newborns [12].

Because of their key function in the initiation and regulation of adaptive immune responses, it is reasonable to assume that antigen presenting cells (APC), such as monocytes and dendritic cells (DC), contribute to the modulation of foetal immune responses upon exposure to *P. falciparum in utero*. Indeed, DC seem to play an important role in both protective and dysfunctional immune responses against malaria in murine models [13,14]. DC comprise a heterogeneous population of cells; myeloid DC (MDC) that orchestrate T-cell responses through a fine modulation of IL-12 secretion, while plasmacytoid DC (PDC) are an essential component of innate and adaptive immunity through secretion of type I interferons (IFN) in response to pathogens [15]. A minor blood MDC population, blood DC antigen (BDCA)-3^+ ^cells, has been described sharing the same ontogeny as the more frequent BDCA-1^+ ^MDC subset [16,17].

The foetal/neonatal immune system exhibits quantitative and functional differences from the adult one and neonatal DC have reduced ability in delivering co-stimulatory signals to T-cells as a consequence of their incomplete maturation [18]. They also exhibit a markedly decreased capacity in secreting IL-12 and IFN-α [19,20]. This probably contributes to the development and relative predominance of Treg in CB [21], although seemingly less marked in Africans vs. Europeans [22].

Whether and how *P. falciparum *infection in the mother may affect foetal innate immunity is poorly understood. One study conducted in The Gambia reported lower lipopolysaccharide (LPS)-induced IFN-γ and IL-12 activity in CBMC of newborns of mothers with PAM as compared to uninfected mothers [9]. A more recent study revealed that CBMC of neonates born of Gabonese mothers with *P. falciparum *infection exhibit significantly increased IFN-γ responses upon stimulation with toll-like receptor (TLR)3 and TLR4 ligands [22].

Contrasting findings have also been reported on the characterization of DC subsets in CBMC of neonates born to *P. falciparum*-infected mothers. One study reported a significantly higher frequency of MDC [23], while another reported profoundly reduced numbers of PDC [24] as compared to unexposed newborns.

The mechanistic hypothesis behind the present study is that malaria infection in the mother may cause a dysfunctional activation of foetal APC by parasite-derived products that cross the placenta. An altered activation of foetal APC could be responsible for the impaired T-cell response that is observed in infants born to mothers with PAM.

Using flow cytometry, subpopulations of DC and monocytes were evaluated in CB of neonates from Beninese women with or without malaria infection. In addition, the impact of *P. falciparum *exposure *in utero*, on the innate activation of foetal APC was examined by stimulating CBMC with specific TLR ligands; LPS was employed to activate TLR4 on monocytes and BDCA-1^+^MDC, polyinosine-polycytidilic acid (PolyI:C) to selectively stimulate TLR3 expressed in MDC, and CpG-A ODN to specifically activate TLR9 expressed in PDC [16,17,25].

## Methods

### Study population

Pregnant women were enrolled after informed consent from July 2006 to January 2007; in the Hospital "Mother and Child Lagune", the main obstetrical referring hospital in Cotonou. This study was approved by the Science and Health Faculty Ethics Committee. To identify women with malaria infection, a rapid immuno-chromatographic test (Cypress^®^, Langdorp, Belgium) was performed on finger-pricked capillary blood before delivery. Thirty *P. falciparum*-infected women and twenty-nine uninfected women matched for parity and age were enrolled in the study. Twenty-five ml heparinized CB were collected immediately after delivery. According to national policy, pregnant women receive intermittent preventive treatment with sulphadoxine-pyrimethamine (SP). Despite this usage of SP was declared by only 47% of the women, while the remaining mothers declared having taken chloroquine (CQ) as chemoprophylaxis.

### Determination of *P. falciparum *status of the mothers at delivery

Thin and thick smears were prepared from maternal peripheral, placental intervillous and cord blood, stained with Giemsa and examined for the presence and density of parasites. Malaria infection in the mothers at delivery was defined by the presence of parasites in the placental and/or maternal peripheral blood. The presence of malaria pigment (MP) was also evaluated in leukocytes of placental intervillous blood (Table 1).

**Table 1:** Summary of the study population.

	Study group	
	
Characteristics	*P. falciparum*-positive	*P. falciparum*-negative
Number of subjects n = 59	30	29
Age of mother, mean ± SD, years	25.4 ± 6.1	26.3 ± 5.2
Pregnancies, no., mean ± SD	2.2 ± 1.5	2.1 ± 1.1
1-2 pregnancies (n = 41)	21	20
≥ 3 pregnancies (n = 18)	9	9
Ratio of malaria prevention (CQ/SP^a^) (30/27)	19/10	11/17
Declaration of malaria infection during pregnancy (%)	33.3	3.7
Reported use of bednet (%)	83.3	82.8
Neonate birth weight, mean ± SD, g	3052.8 ± 443.3	3059.6 ± 412.5
Neonate gender, female/male (21/34)	10/19	11/15
*P. falciparum *density at delivery:		
peripheral blood, mean ± SD, iRBC^b^/μl	19,037 ± 55,257	0
intervillous blood, mean ± SD, iRBC/μl	245,764 ± 475,906	0
cord blood, mean ± SD, iRBC/μl	0	0
intervillous blood leukocytes with MP^c^, n = 59	19	0

^a ^CQ/SP; Chloroquine/sulfadoxine-pyrimethamine

^b ^iRBC; infected red blood cells

^c ^MP; malaria pigment

### CBMC cultures

Mononuclear cells were isolated from CB by centrifugation over Ficoll-Hypaque (Pharmacia Uppsala, Sweden). Cells were washed twice and resuspended in RPMI 1640 medium with L-glutamine (Gibco Eragny, France) supplemented with 10% foetal bovine serum (FBS, Gibco) and 50 μg/ml gentamycin to a final concentration of 2 × 10^6 ^CBMC/ml. Viability was > 99% in all tested samples as determined by Trypan blue staining.

To assess production of IL-12, CBMC were stimulated for 8 hours with LPS (100 ng/ml; Sigma Aldrich, St. Louis MO), PolyI:C (20 μg/ml; Sigma Aldrich), or synthetic haemozoin (Hz, 5 μg/ml) in the presence of Brefeldin-A (BD Pharmingen, San Diego, CA) during the last five hours of incubation. Hz was prepared from haemin chloride as described [26]. Endotoxin levels in the Hz preparation were found to be below the threshold (<0.125 units/ml) by the Limulus-amoebocyte lysate assay (Biowhittaker, Cambrex). To assess cytokine production by CBMC upon contact with TLR9 ligands, CpG-A ODN 2216 (3 μg/ml; Metabion GmbH, Martinsried, Germany) was employed.

### Immunophenotype of APC

**Figure 1:**
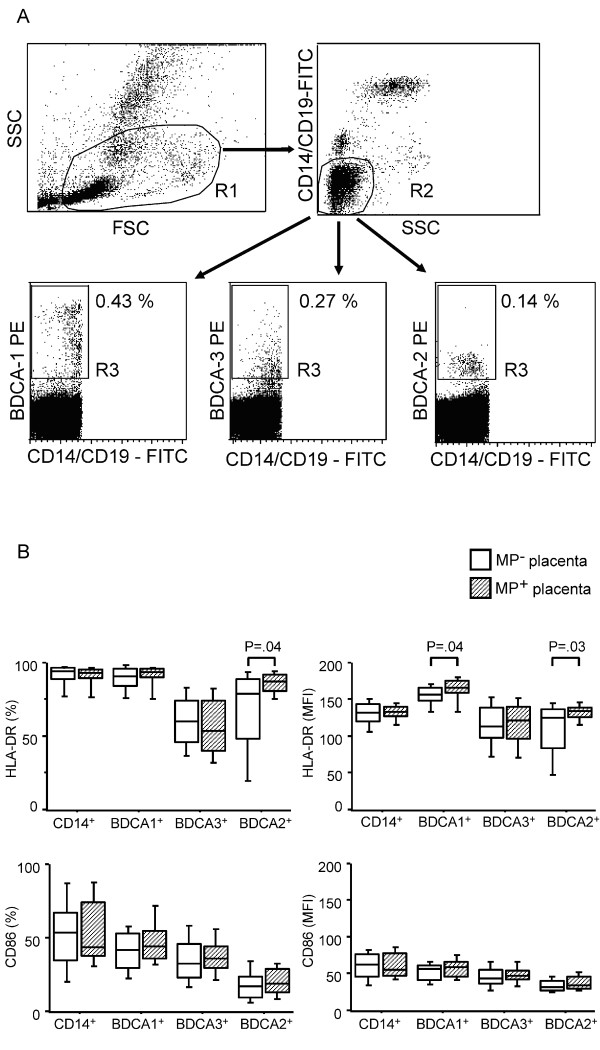
**(A) Flow cytometric identification of MDC (BDCA-1^+ ^or BDCA-3^+^) and PDC (BDCA-2^+^) in CBMC from one subject**. R1 gate was set to include only viable mononuclear cells, as determined by forward scatter (FSC) and side-scatter (SSC) characteristics. Furthermore, cells were gated to be both CD14 and CD19 negative (R2) and either BDCA-1, BDCA-3, or BDCA-2 positive (R3). One million events were analysed for DC, 300,000 events for monocytes and 100,000 events for isotype control enumeration. The percentages represent relative levels of the different DC populations from the selected subject. (B) Partial activation of cord blood BDCA-1^+ ^and BDCA-2^+ ^cells is related to the presence of MP in placenta. The expression levels of HLA-DR and CD86 were measured by flow cytometry on foetal APC subsets from 16 MP-positive (diagonal striped bars) and 39 MP-negative mothers (white bars). Boxplots illustrate the medians and the 25^th ^and 75^th ^percentiles. Y-axes report percentage of positive cells and mean fluorescence intensity (MFI) for the activation markers CD86 and HLA-DR in different APC subsets. P-values were calculated by Mann-Whitney U test. The significance limit was P < 0.05.

CBMC were resuspended in staining buffer (PBS 2% FBS, 5 mM EDTA). Cells were first incubated with FcR blocking reagent (Miltenyi Biotech, Bergisch-Gladbach, Germany) and then with anti-CD14-FITC, anti-CD19-FITC, anti-BDCA-1-PE, anti-BDCA-2-PE, anti-BDCA-3-PE (Miltenyi Biotech), anti-HLA-DR-PerCP and anti-CD86-APC (BD Pharmingen), or alternatively mouse isotype controls (BD Pharmingen). Cell acquisition was performed with a FACSCalibur flow cytometer (BD Pharmingen) and analysis was performed by CellQuest software as described in Figure 1A.

### Intracellular cytokine staining for IL-12

After stimulation, cells were incubated with FcR blocking reagent and stained with anti-HLA-DR-PerCP, anti-CD14-FITC, anti-CD19-FITC, anti-BDCA-1-PE, anti-BDCA-3-PE or isotype controls for 10 min at 4°C. Cells were then fixed with FACS lysing solution, washed and incubated in a permeabilization buffer (staining buffer with 0.25% saponin and 5% AB human serum) for 15 min at 4°C. After centrifugation, cells were stained with anti-IL-12-APC (BD Pharmingen) or alternatively APC-conjugated isotype control for 30 min at 4°C, and then analysed by flow cytometry. BDCA-1^+ ^and BDCA-3^+ ^cells that did not express CD19 and CD14 were gated together as MDC.

### Determination of cytokine levels in plasma samples and supernatants

IFN-α levels were measured with an ELISA kit (PBL Biomedical Laboratories, Piscataway, NJ). The assay sensitivity was 12.5 pg/ml. A panel of pro-inflammatory and anti-inflammatory cytokines including IL-6, IL-10, IL-12, (MIP)-1α/CCL3; TNF-α and IFN-γ were quantified by the Human Cytokine Cytometric Bead Array Kit (BD Pharmingen) using flow cytometry. The assay sensitivity was 1.6 pg/ml; 0.13 pg/ml; 0.6 pg/ml; 0.2 pg/ml; 1.2 pg/ml; and 1.8 pg/ml for IL-6, IL-10, IL-12, MIP-1-α/CCL3, TNF-α and IFN-γ respectively. Results were formatted using the BD CBA Analysis Software.

### Statistical analysis

Background values on cytokines in supernatants obtained from unstimulated cells were subtracted from data acquired from cultures in the presence of stimuli. Normally distributed variables were analysed by unpaired *t *test. Data that were not normally distributed even after log-transformation were analysed by the non-parametric Mann-Whitney test. To test if the age of the mother was related to parity, Spearman rank correlation was employed. Linear regression analysis on log-transformed data was used to identify dependent variables for a multivariate analysis. The significance limit was P < 0.05.

## Results

### Partial activation of foetal DC is related to the presence of MP in placenta

The absolute numbers of APC subpopulations and expression of activation markers are described in Table 2. CBMC contained MDC at a higher frequency than PDC, resulting in a mean BDCA-1^+^/BDCA-2^+ ^cell ratio of 4.4 ± 5.2.

**Table 2:** APC absolute numbers and immunophenotype in cord blood samples.

	APC subset			
	
Parameter: median (± interquartile)	Monocytes	MDC		PDC
	**CD14^+^**	**BDCA-1^+^**	**BDCA-3^+^**	**BDCA-2^+^**
	
% of total CBMC	13.00 ± (7.10)	0.62 ± (0.42)	0.19 ± (0.18)	0.25 ± (0.18)
absolute no./ml	624,787 ± (571,630)	23,850 ± (30,430)	8,160 ± (12,225)	9,680 ± (9,032)
% HLA-DR^+ ^cells	93.87 ± (7.09)	93.53 ± (11.01)	59.95 ± (28.55)	82.64 ± (31.69)
HLA-DR, MFI^a^	132.71 ± (17.69)	162.50 ± (18.81)	118.26 ± (40.81)	129.9 ± (39.59)
% CD86^+ ^cells	50.28 ± (34.37)	44.67 ± (22.15)	35.95 ± (20.98)	18.47 ± (13.41)
CD86, MFI	62.36 ± (30.17)	57.51 ± (21.12)	45.54 ± (17.80)	33.29 ± (13.77)

Immunophenotyping was performed on 55 cord blood samples; 27 from malaria infected women and 28 from uninfected women. APC were quantified as a percentage of the total CBMC. Absolute numbers of MDC, PDC and monocytes were calculated from CBMC counts. HLA-DR and CD86 expression levels were then examined in different APC subsets.

^a^; MFI, mean fluorescence intensity

Segregation on the basis of maternal malaria infection (i.e. presence of parasites in placental and/or maternal peripheral blood) did not show differences either in the absolute number of foetal APC or in the expression levels of MHC class II and CD86 molecules in different APC subsets studied (additional file 1). However, foetal APC status segregated on the basis of presence or absence of MP in the placenta, revealed a significant up-regulation of the MHC-class II expression, but not of CD86, on BDCA-1^+ ^and BDCA-2^+ ^DC in CB obtained from MP-positive mothers as compared to MP-negative mothers (Figure 1B). Thus, a partial activation of foetal DC is related to the presence of MP in the placenta, and not to maternal infection at delivery.

### Impact of age and parity of the mother on the frequency and activation status of foetal APC

**Figure 2:**
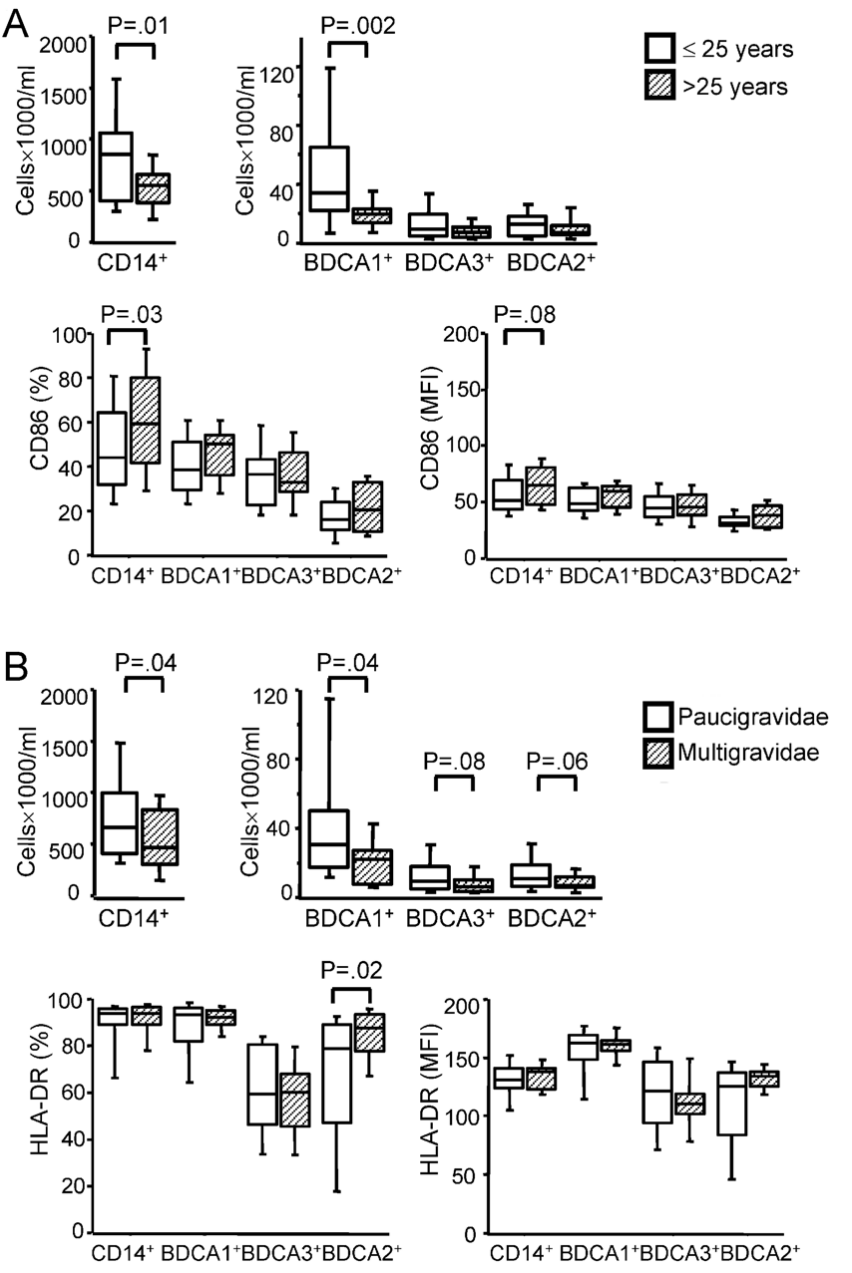
**Association between APC numbers and activation and maternal age or parity**. (A) Data were segregated into 2 groups according to the median value for maternal age. Absolute numbers and CD86 expression levels (as percentage of positive cells and mean fluorescence intensity, MFI) on different foetal APC subsets were analysed as a function of maternal age in 31 women ≤ 25 years of age and 24 women > 25 years of age Boxplots illustrate the medians and the 25^th ^and 75^th ^percentiles. (B) Women were divided for parity as paucigravidae (1st and 2nd pregnancy) and multigravidae (≥ 3 pregnancies) as we previously observed that women at first and second pregnancy exhibited the same risk of malaria infection (Fievet, unpublished data). Absolute numbers and HLA-DR expression levels (as percentage of positive cells and mean fluorescence intensity, MFI) on different foetal APC subsets were analysed as a function of parity in 37 women undergoing first or second pregnancy and 18 multigravidae. Boxplots illustrate the medians and percentiles. P-values were calculated by Mann-Whitney U test. The significance limit was P < 0.05.

The absolute numbers of monocytes and BDCA-1^+ ^MDC in CB were negatively associated with maternal age and parity (Figure 2A and 2B). In addition, maternal age showed a positive correlation with higher expression levels of CD86 on monocytes (Figure 2A). We also observed significantly increased MHC-class II expression on BDCA-2^+ ^DC in CB from multigravidae as compared to mothers undergoing first or second pregnancy (Figure 2B).

As expected, maternal age and multiparity were related (Spearman coefficient = 0.47; P = 0.0002). The multivariate analysis showed an effect of both age and parity on CB monocytes and BDCA-1^+ ^cells absolute numbers (age: β = +0,55 (0,10-1,00) with P = 0,02 and parity: β = +0,42 (-0,06-0,90) with P = 0,08).

### Malaria status of the mother does not affect immunophenotype of foetal APC upon TLR and Hz stimulation

By examining MHC class II expression, CB monocytes and MDC were activated by LPS and PolyI:C stimulation (Table 3). In addition, when we added synthetic Hz as stimulus, we found monocytic activation, as shown by significantly increased expression levels of MHC class II molecules, while the MHC class II levels on MDC were not affected (Table 3). However, there was no difference in MHC class II expression following stimulation with TLR3 and TLR4 ligands or synthetic Hz on CB monocytes and MDC when segregated according to the presence of MP in the placentas (Table 3). Thus, MP positivity did not affect further phenotypic *ex- vivo *maturation of foetal APC by diverse TLR ligands or Hz.

**Table 3:** Immunophenotype of fetal APC upon TLR and Hz stimulation.

			Monocytes			MDC	
		
Parameter median (± interquartile)	stimulus	Total CB	MP-Negative	MP-Positive	Total CB	MP -Negative	MP-Positive
% HLA-DR^+ ^cells	unstimulated	89.34 ± (13.90)	89.33 ± (16.57)	89.79 ± (7.27)	88.78 ± (19.83)	87.33.40 ± (19.83)	92.62 ± (20.13)
	Hz	91.03 ± (10.10)	90.46 ± (11.97)	92.27 ± (8.31)	90.99 ± (12.22)	89.40 ± (13.55)	91.37 ± (7.88)
	LPS	96.73 ± (6.22)	96.73 ± (7.61)	96.45 ± (3.14)	93.24 ± (11.08)	93.24 ± (11.66)	93.38 ± (15.23)
	Poly I:C	96.79 ± (4.68)	96.79 ± (7.99)	96.70 ± (2.27)	93.99 ± (12.57)	93.08 ± (9.54)	92.09 ± (16.42)

HLA-DR, MFI^a^	unstimulated	120.81 ± (20.14)	121.15 ± (24.36)	119.54 ± (16.48)	143.85 ± (23.19)	143.57 ± (21.28)	149.29 ± (22.77)
	Hz	122.08 ± (20.42) *	125.36 ± (23.00)	121.47 ± (15.15)	146.96 ± (14.93)	151.30 ± (14.69)	156.06 ± (20.47)
	LPS	135.72 ± (16.78)*	137.56 ± (18.02)	133.23 ± (13.82)	152.40 ± (16.28)*	145.49 ± (19.31)	150.67 ± (13.84)
	Poly I:C	136.53 ± (15.66)*	137.27 ± (19.29)	136.41 ± (13.51)	150.16 ± (14.93)*	150.61 ± (16.72)	149.60 ± (16.35)

Immunophenotyping on stimulated cells was performed on 43 cord blood samples (total CB); 12 from women with MP accumulation in placenta (MP-Positive) and 31 without MP in placenta (MP-negative). All comparison between MP-Negative and MP-Positive were not significant.

^a^; MFI, mean fluorescence intensity

*; P < 0.05

### *Plasmodium falciparum *infection in the mother induces amplification of TLR9 response in foetal leukocytes

Cytokine secretion in APC is triggered by the recognition of microbial pathogens through TLR [15]. The ability of foetal APC to secrete cytokines by stimulating CBMC with different TLR ligands was investigated. IFN-α was evaluated in supernatants of unstimulated, CpG-A- and Hz-stimulated CBMC cultures. As previously reported [19,27], unstimulated and synthetic Hz-stimulated CBMC did not secrete any IFN-α, while CpG-A-stimulated cells, most likely PDC, produced low levels of this cytokine (0.12 ± 13.79 pg/ml; median ± interquartile). No difference was observed in IFN-α production upon CpG-A stimulation when CBMC were segregated according to accumulation of MP in placenta: (0.01 ± 12.04 pg/ml vs 1.90 ± 18.21 pg/ml; MP-negative vs MP-positive women; p = 0.45).

**Figure 3:**
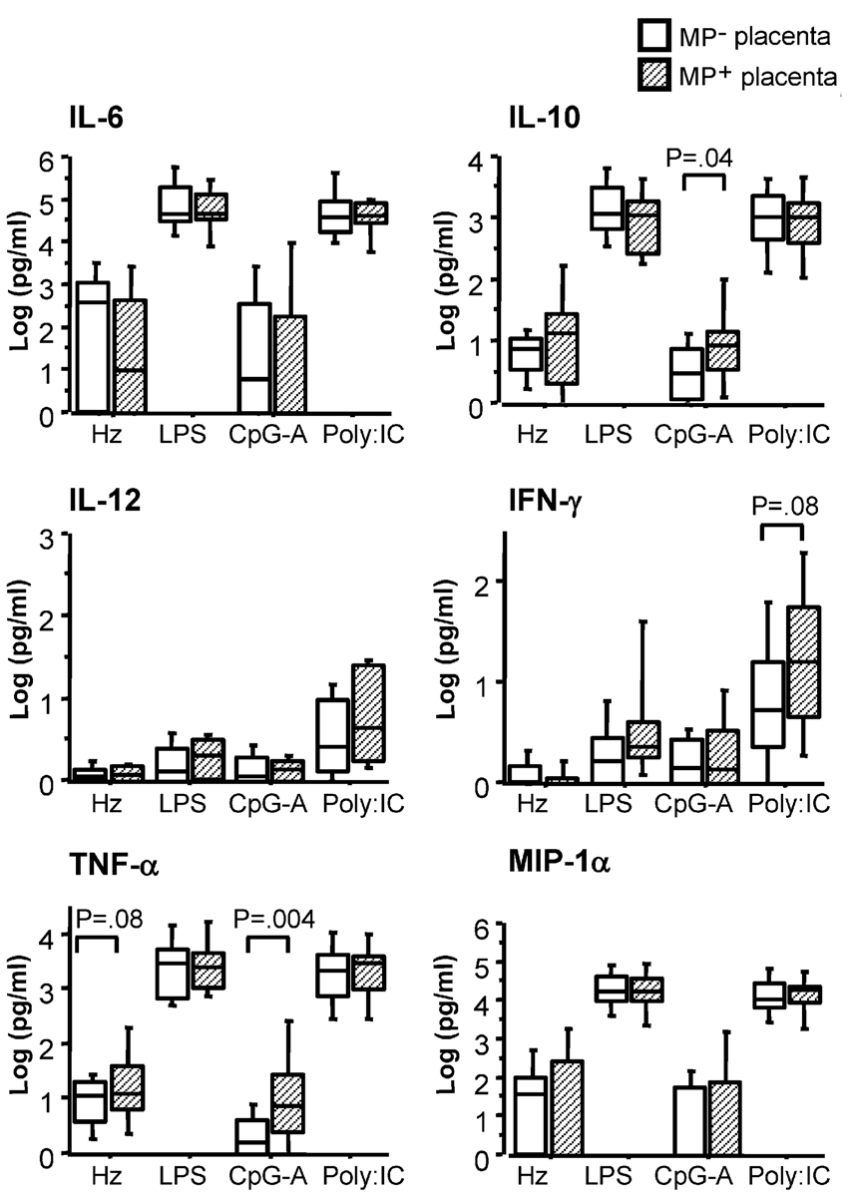
**TLR-induced cytokine responses in CBMC obtained from women with or without MP accumulation in placenta**. CBMC (2 million/ml) were stimulated or not with synthetic Hz (5 μg/ml), LPS (100 ng/ml), CpG-A (3 μg/ml) or PolyI:C (20 μg/ml). After 18 hours, supernatants were collected and analysed for IL-6, IL-10, IL-12, IFN-α, TNF-α and MIP-1α/CCL3 levels. Data represent median values and percentiles for 59 individuals; 16 MP-positive (diagonal striped bars) and 43 MP-negative mothers (white bars). Cytokine levels in unstimulated cells were subtracted from the values shown. P-values were calculated by Mann-Whitney U test or t test (see Subjects, Materials and Methods). The significance limit was P < 0.05.

The levels of IL-6, IL-10, IL-12, MIP-1α/CCL3α IFN-γ and TNF-α were also quantified in supernatants of CBMC that were either unstimulated or stimulated with synthetic Hz or different TLR ligands. TLR3, TLR4 and, to a lesser extent, TLR9 stimulation induced release of both pro- and anti-inflammatory cytokines (Figure 3). The presence of MP in the placenta influenced TLR-mediated CBMC cytokine responses such that the production of IL-10 and TNF-α was significantly higher upon TLR9 stimulation and there was a similar trend for increased production of IFN-γ after TLR-3 stimulation in CBMC of neonates of mothers with MP-positive placenta as compared to MP-negative mothers (Figure 3).

To further analyse the production of IL-12 by different APC subsets upon TLR3 and TLR4 stimulation, we performed intracellular cytokine staining. Only a minor population of foetal APC produced IL-12. In fact, 2.7% ± 3.4% and 2.6% ± 3.8% of IL-12-producing monocytes (median ± interquartile) were observed in response to LPS and PolyI:C, respectively, and 2.7% ± 3.5% and 2.0% ± 3.6% of IL-12-producing MDC (median ± interquartile) were found in responses to LPS and PolyI:C, respectively. However, no differences were observed when comparing foetal APC of mothers with or without MP accumulation in placenta (table 4).

**Table 4:** Accumulation of MP in placenta does not affect IL-12 production by APC upon TLR3 and TLR4 stimulation.

		Monocytes		MDC	
		
Parameter median (± interquartile)	stimulus	MP-Negative	MP-Positive	MP-Negative	MP-Positive
IL-12 (% positive cells)	LPS	3.55 ± (4.13)	2.70 ± (3.15)	3.13 ± (3.72)	2.13 ± (1.72)
	Poly:IC	2.33 ± (3.37)	3.99 ± (4.49)	1.95 ± (4.01)	2.38 ± (3.09)

Intracytoplasmic IL-12 production by APC upon TLR3 and TLR4 stimulation was analysed on 43 cord blood samples; 31 from MP-negative women and 12 MP-positive women. All comparisons between MP-negative and MP-positive women were not significant.

**Figure 4:**
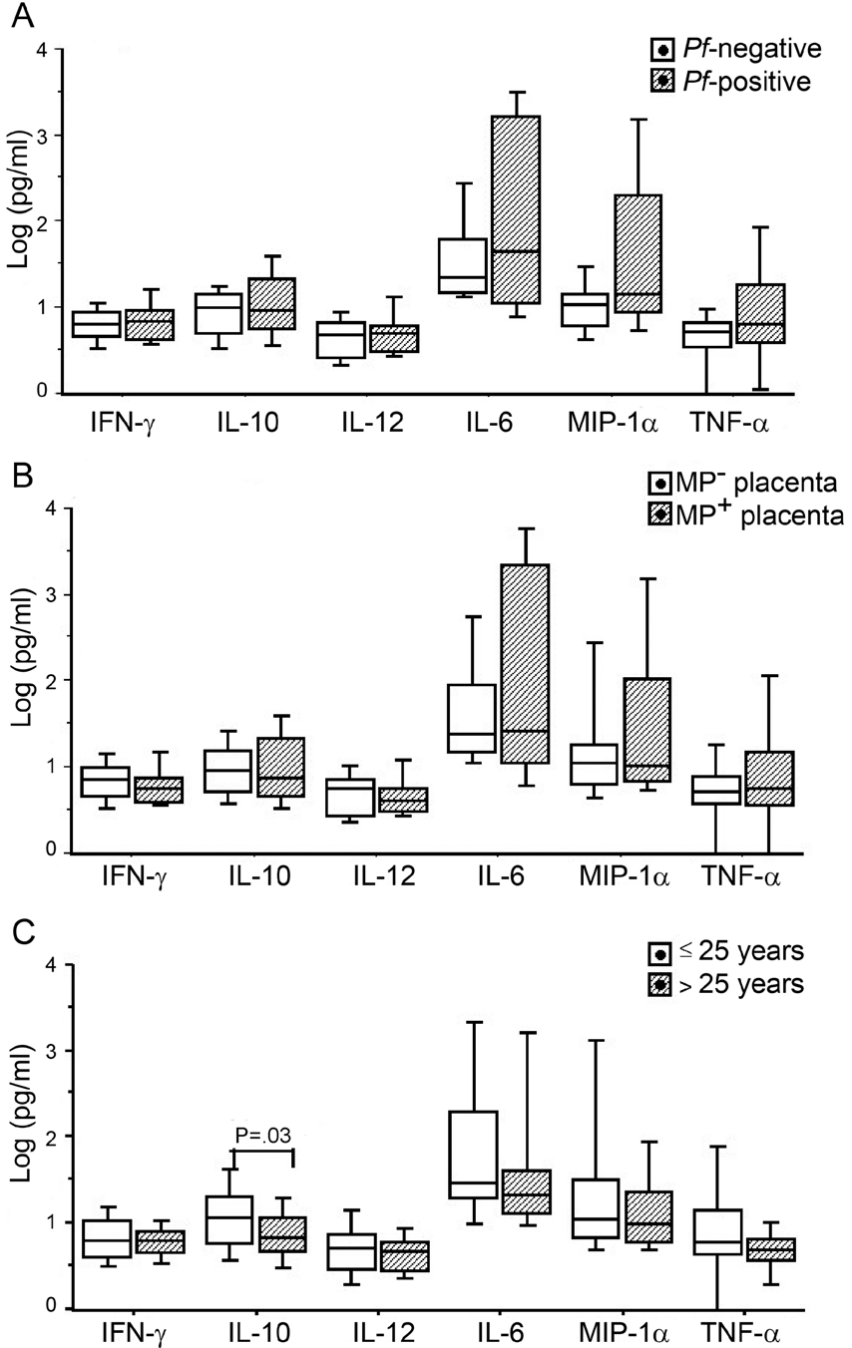
**Cord blood cytokine levels are affected by maternal age, but not by maternal P. falciparum infection or MP accumulation in placenta**. Foetal plasma samples were collected at delivery and subsequently analysed for IL-6, IL-10, IL-12, IFN-γ, TNF-α and MIP-1α/CCL3 levels. (A) Data represent median values and percentiles for 57 individuals divided in 27 *P. falciparum *negative mothers (Pf-negative, white bars) and 30 *P. falciparum *positive mothers (Pf-positive, diagonal striped bars). (B) Women were divided in 39 MP-negative mothers (white bars) and 18 MP-positive (diagonal striped bars). (C) Women were divided in 33 mothers ≤ 25 years of age (white bars) and 24 women > 25 years (diagonal striped bars) Values were calculated by Mann-Whitney U test or t test (see Subjects, Materials and Methods). The significance limit was P < 0.05.

Fifty-seven plasma samples from CB of mothers were analysed for cytokine levels. In all samples we found detectable levels of IFN-α, IL-6, IL-10, IL-12, MIP-1-α/CCL3 and TFN-γ but no IFN-γ. The CB plasma levels of the different cytokines were not influenced by the presence of malaria infection in the mother (Figure 4A) or by MP accumulation in placenta (Figure 4B).

Thus, cytokine responses to TLR3, 4 and 9 ligands and to Hz were observed, with amplification of TLR9-mediated responses in CBMC from MP-positive mothers.

### Maternal age influences TLR3, 4 and 9 responses of foetal leukocytes and cytokine levels in foetal plasma

**Figure 5:**
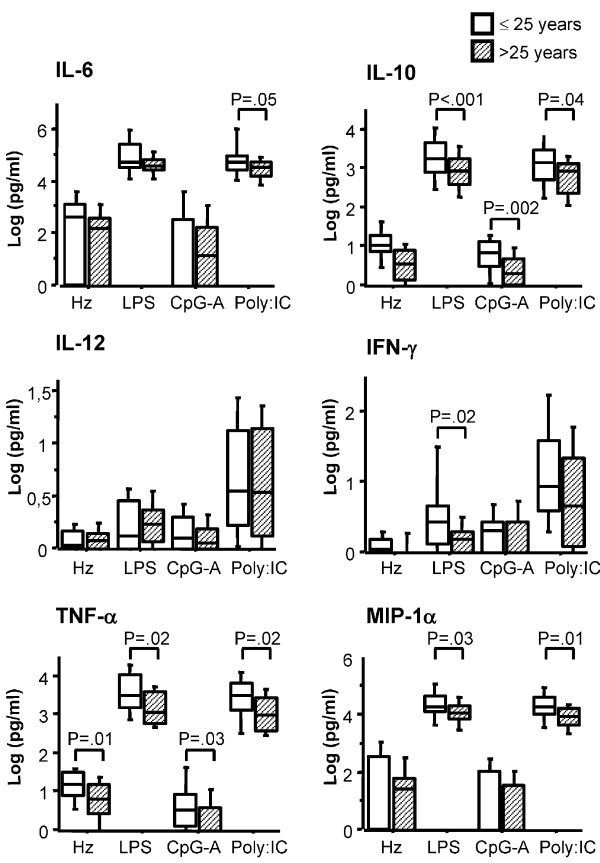
**TLR-induced cytokine responses in foetal leukocytes are influenced by maternal age**. CBMC (2 million/ml) were stimulated with synthetic Hz (5 μg/ml), LPS (100 ng/ml), CpG-A (3 μg/ml) or PolyI:C (20 μg/ml). After 18 hours, supernatants were collected and analysed for IL-6, IL-10, IL-12, IFN-α, TNF-α and MIP-1α/CCL3 levels. Cytokine levels in unstimulated cells were subtracted from the values shown. P-values were calculated by Mann-Whitney U test or t test (see Subjects, Materials and Methods). The significance limit was P < 0.05. Data represent median values and percentiles for 57 individuals; 33 mothers ≤ 25 years of age (white bars) and 24 women > 25 years (diagonal striped bars) (Data on maternal age were missing for 2 subjects, that were therefore not included in this analysis).

**Figure 6:**
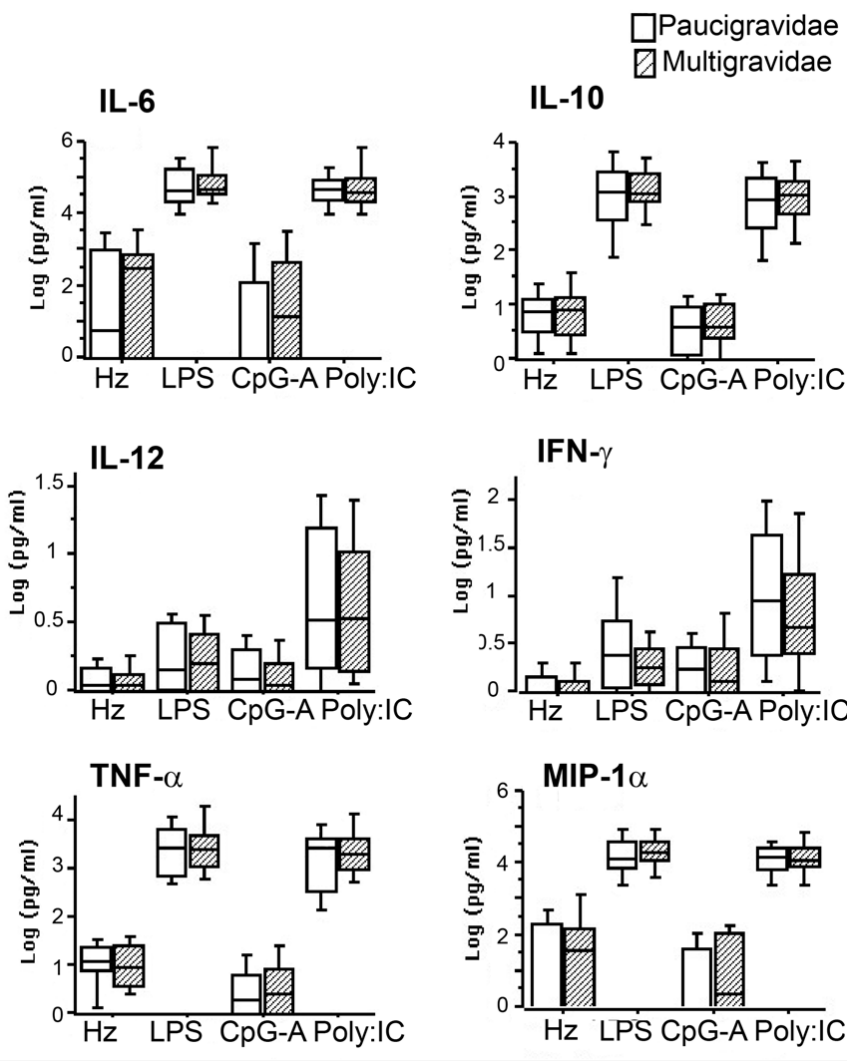
**TLR-induced cytokine responses in foetal leukocytes are not influenced by parity**. CBMC (2 million/ml) were stimulated with synthetic Hz (5 μg/ml), LPS (100 ng/ml), CpG-A (3 μg/ml) or PolyI:C (20 μg/ml). After 18 hours, supernatants were collected and analysed for IL-6, IL-10, IL-12, IFN-α, TNF-α and MIP-1α/CCL3 levels Cytokine level in unstimulated cells was subtracted from the values shown. P-values were calculated by Mann-Whitney U test or t test (see Subjects, Materials and Methods). The significance limit was P < 0.05. Data represent median values and percentiles for Women were segregated on the basis of parity as 41 paucigravidae (1st and 2nd pregnancy; white bars) and 18 multigravidae (≥ 3 pregnancies; striped bars).

Segregation of samples into two groups according to the median value for maternal age revealed significant differences in cytokine production after TLR3, 4 and 9 stimulation of CBMC. The levels of TNF-α, IFN-γ, MIP-1α/CCL3 and IL-10 produced in response to LPS by CBMC of newborns of mothers ≤ 25 years were significantly higher than those produced by CBMC of neonates born to mothers > 25 years. In addition, the amount of TNF-γ and IL-10 produced by CBMC in response to TLR3 and TLR9 ligands were negatively associated with maternal age (Figure 5). Parity had no influence on cytokine secretion by CBMC upon TLR stimulation (Figure 6).

Significant differences were also observed in CB cytokine levels according to maternal age. Foetal plasma from mothers > 25 years of age exhibited significant lower levels of IL-10 as compared to younger mothers (Figure 4C). Thus, productions of cytokines by CBMC and plasma levels for IL-10 were higher in children born to the younger mothers.

When an effect of both MP-positivity in placenta and maternal age on cytokines in supernatants was observed, the multivariate analysis showed that maternal age was predominant and that effect of MP-positivity disappeared for IL-10 in response to TLR9 ligands (β = 1.23 (0.56-1.89) P = 0.001). However, both MP-positivity and maternal age had an effect on TNF-α secretion upon TLR9 stimulation (MP: β = 0.88 (0.024-1.74), P = 0.04; Age: β = 1.04 (0.23-1,74), P = 0.04).

## Discussion

The consensus view is that *in utero *sensitization to *P. falciparum *antigens is a common phenomenon during PAM. Parasites do not usually cross the placental barrier and such sensitization is most probably caused by the transplacental passage of soluble *P. falciparum *antigens [8,28]. Accordingly, no parasite-positive smears were detected in CB samples of malaria-infected women in this study population.

Pregnant women declared to receive either SP or CQ as malaria prevention during pregnancy. In contrast to a previous study performed in the same area [29], no differences were observed for *P. falciparum *infection rate according to the type of prophylaxis used by the mothers. However, the number of subject included in this study was low and the project was not designed to examine this matter.

In this study, the foetal BDCA-1^+ ^and BDCA-2^+ ^DC subsets expressed significantly higher levels of MHC class II molecules upon PAM, as indicated by the presence of *P. falciparum *MP in placenta, which is in agreement with previous data [23]. The observation that CD86 expression on foetal DC was unaffected by PAM suggests that *P. falciparum *stimulation *in utero *induces only partial activation of these cells. Failure to provide DC with a sufficiently strong costimulatory signals can impair the ability to form stable interactions with T-cells, as recently shown in a murine model of malaria [30]. Partial DC maturation can lead to altered T-cell activation and induction of tolerance [31-34], possibly contributing to impaired immune responses that have been observed in the offspring of mothers with PAM [9,11].

The findings presented in this study diverge from those of studies on peripheral blood DC from children with acute malaria, where expression levels of MHC class II on the BDCA-1^+ ^DC are reduced compared to healthy controls [35,36]. Also, no increase in foetal BDCA-3^+ ^DC was detected upon maternal malaria infection in this study like others have shown in children with severe malaria [35]. Circulating APC are continuously exposed to *P. falciparum*-infected erythrocytes during malaria episodes in children, which may exert a contact-mediated inhibitory effect on DC functionality, as demonstrated by *in vitro *studies [4,37]. Conversely, infected erythrocytes are rarely detected in CB of those born to mothers with PAM [23,38]. Thus, foetal APC would rarely if ever encounter parasitized red-blood cells, but would be primarily exposed to and influenced by parasite-derived soluble compounds.

Interestingly, TLR9 stimulation led to increased pro- and anti-inflammatory responses of CBMC of neonates whose mothers had MP accumulating in placentas, and there was a tendency towards increased IFN-γ response upon TLR3 stimulation in the same group. Responses *via *other TLR ligands, such as LPS were amplified in CBMC but did not change appreciably as a function of maternal malaria infection. Thus, foetal different TLR responses are independently modulated by *in utero *exposure to *P. falciparum*, consistent with a recent study [22].

In humans only PDC and B cells express TLR9 [39]. In this study, CpG-A, a TLR9 ligand that specifically stimulates PDC [25,40], was employed. In concordance with the findings presented in this study, MP or alternatively plasmodial DNA bound to MP activate the TLR9 pathway in human and murine PDC [27,41,42]. This would suggest a role for MP derived from maternal parasitic infection in inducing foetal BDCA-2^+ ^DC partial maturation and increased sensitization to TLR9 ligands. Nevertheless, only low levels of IL-10 and TNF-α were detected in CBMC cultures upon TLR9 stimulation. This was not unexpected given the low frequency of cells able to specifically respond to such stimulus. The biological significance of a slightly increased release of IL-10 and TNF-α by CBMC upon TLR9 stimulation after *in utero *exposure to *P. falciparum *is uncertain.

Notably, MP was the only indicator of maternal malaria infection that was significantly associated with partial activation of foetal DC and to amplified innate response to TLR9 ligation, while other markers of maternal parasitization at delivery such as the presence of parasites in peripheral and/or placental blood, were unrelated to DC activation in the exposed newborns. It has been recently postulated that accumulation of MP in leukocytes is a good indicator of total parasite burden, including parasite sequestration [43], and therefore we can consider accumulation of MP in placenta as a marker of high intensity of maternal malaria infection and/or of prolonged parasite exposure. In addition, accumulation of MP in placental leukocytes has been associated with increased monocyte activation and inflammation [44]. As a hypothesis, accumulation of MP may represent a specific activation stimulus and inflammation at the placental level and this may cause partial and inadequate activation of APC in the foetal compartment.

Additionally, maternal age and parity should be taken into consideration when analysing foetal/neonatal innate immunity. Women of higher parity and increased age delivered babies in whom significantly fewer blood APC were found, but these cells exhibited an enhanced activation status. Maternal age but not parity also influenced the APC cytokine responses upon TLR stimulation, such that CBMC of offspring of younger mothers exhibited an increased ability to respond to TLR3, 4 and 9 ligands. These data are in agreement with published data on African [23] and Caucasian [45] women and suggest that maternal age and obstetric history may influence foetal/neonatal immune parameters.

Consequences of increased maternal age and/or multiple parities in terms of neonatal responses to pathogens are poorly understood. Two recent studies indicate that the frequency of malaria episodes is higher among infants of malaria-infected multigravidae as compared to primigravidae [6,7]. The intrinsic effect of multiple pregnancies on malaria susceptibility in the offspring may be at least partially explained by our finding of a significantly reduced number of myeloid APC in foetal blood from multigravidae. How maternal age or alternatively parity can affect the number, activation status and cytokine secretion capacity of cord blood APC is presently unknown.

In conclusion, placental parasitization, as indicated by the presence of MP in placental leukocytes, is significantly associated with partial maturation of different DC subsets and to slightly increased responses to a TLR9 ligand in cord blood. As semi-maturation of DC leads to tolerance [46], such partial foetal APC activation may contribute to the altered T-cell responses often observed in newborns of mothers with PAM [5-7].

These observations advocate a possible mechanism by which PAM may modulate foetal/neonatal innate immunity. Further evaluation of APC activation and downstream T-cell responses is ongoing in a large cohort of newborns and infants from mothers with PAM to assess the impact of altered DC activation on the neonatal cell-mediated immunity.

As it is known that neonatal immune responses are largely dependent on the innate branch of immunity and can be improved through selective TLR stimulation [47,48], our results should be considered in the development of effective vaccine strategies for infants living in areas where malaria is endemic.

## Conflict of interests

The authors declare that they have no competing interests.

## Authors' contributions

NF: conceived the study, participated in its design and coordination, acquired the data and contributed to data analysis and interpretation and drafted the manuscript. SV: participated in the design and coordination of the study, contributed in data analysis and interpretation and drafted the manuscript. IS: acquired the data and contributed to their analysis and interpretation. VB: contributed in interpretation and statistical analysis of data and in critically revising the manuscript. SL: contributed in performing the immunoassays, and contributed in their interpretation, participated in critically revising the manuscript. RP: participated in the design of the study, in the analysis of the data and in critically revising the manuscript. AM: participated in the design and coordination of the study and in critically revising the manuscript. AH: participated in the design of the study, in the analysis of the data and in critically revising the manuscript. MT: participated in the design of the study, in the analysis and interpretation of data and in critically revising the manuscript. PD: participated in the design and coordination of the study and contributed to draft the manuscript. All authors read and approved the final manuscript.

## Supplementary Material

Additional file 1 **Presence of *P. falciparum *parasite in maternal and/or placental blood at delivery does not influence the activation status of cord blood DC and monocytes**. The expression levels of HLA-DR and CD86 were measured by flow cytometry on foetal APC from 27 *P. falciparum*-positive (diagonal striped bars) and 28 *P. falciparum*-negative mothers (white bars). Click here for file
