# Supplementary figures and images for: Plasmodium falciparum exposure in utero, maternal age and parity influence the innate activation of foetal antigen presenting cells
Source: Malar J. 2009 Nov 5;8:251. doi: 10.1186/1475-2875-8-251 (PMC2780449; doi:10.1186/1475-2875-8-251)

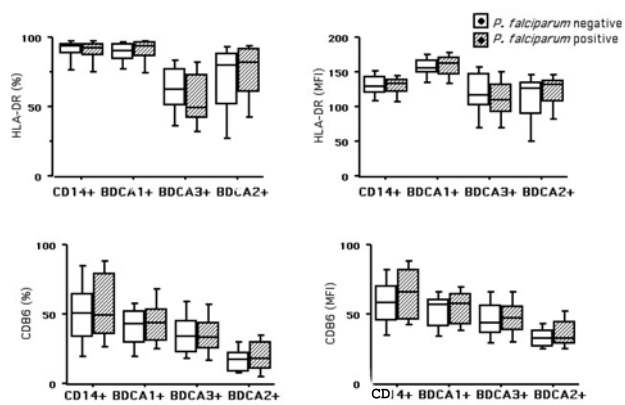

Supplement: Additional file 1 — Presence of P. falciparum parasite in maternal and/or placental blood at delivery does not influence the activation status of cord blood DC and monocytes. The expression levels of HLA-DR and CD86 were measured by flow cytometry on foetal APC from 27 P. falciparum-positive (diagonal striped bars) and 28 P. falciparum-negative mothers (white bars). [file 1475-2875-8-251-S1.pdf]
